# Supplementary material for: Occurrence of Methicillin-Resistant Coagulase-Negative Staphylococci (MRCoNS) and Methicillin-Resistant Staphylococcus aureus (MRSA) from Pigs and Farm Environment in Northwestern Italy
Source: Antibiotics (Basel). 2021 Jun 5;10(6):676. doi: 10.3390/antibiotics10060676 (PMC8227741; doi:10.3390/antibiotics10060676)
Supplement: Supplementary file 1 [file antibiotics-10-00676-s001.zip › antibiotics-1233104-supplementary.pdf]

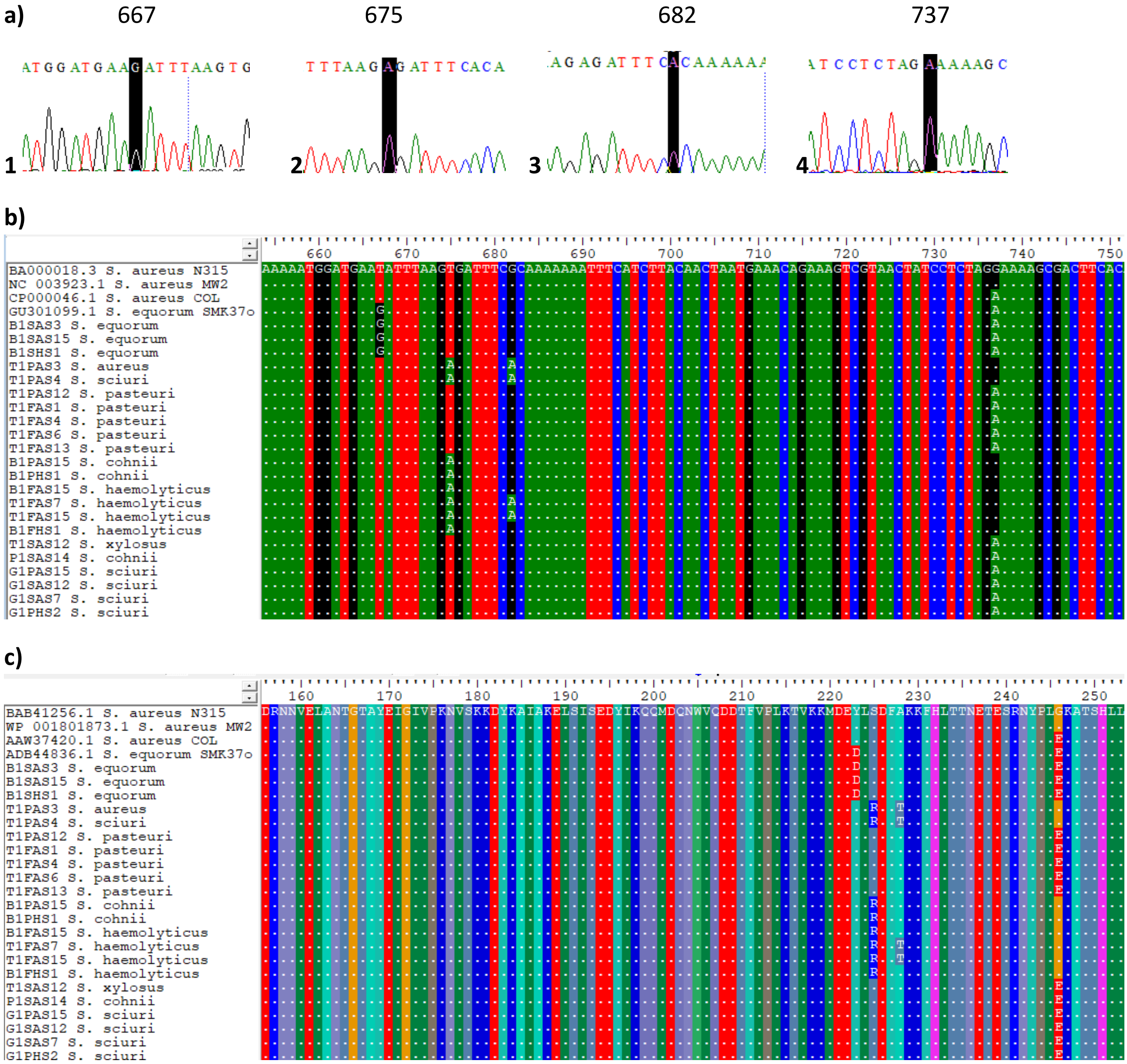

Figure S1. a) Chromatograms of the nucleotide mutations detected in the 527 bp *mecA* fragment: 1) T667G. **2)** T675A. **3)** G682A. **4)** G737A.

b) Multiple nucleotide sequences alignment of a sample (n=22) of methicillin-resistant staphylococci with the four detected mutations. The reference strains (*S. aureus* N315, *S. aureus* MW2, *S. aureus* COL, *S. equorum* SMK37o) are located at the top of the nucleotide alignment.

c) Multiple aminoacidic sequences alignment of a sample (n=22) of methicillin-resistant staphylococci with the four aminoacidic substitutions (Y223D, S225R, A228T, G246E). The reference strains (*S. aureus* N315, *S. aureus* MW2, *S. aureus* COL, *S. equorum* SMK37o) are located at the top of the nucleotide alignment. Chromatograms, nucleotide and aminoacidic multiple alignments were created using BioEdit 7.2.5 Sequence Alignment Editor© software.

Farmers’ questionnaire about biosecurity and general management

Farm ID:

1) Productive cycle: a) open

b) close

c) only finishing

2) Remount: a) internal

b) external: how many gilts’ suppliers do you have?

3) Are new animals quarantined (gilts or new weaned for finishers’ only farm)?

4) Animal mixing a) Is the animals’ flow unidirectional?

b) Are herds composed only by animals from the same group?

c) During which productive phase do you mix animals from different groups?

5) Are farm workers using dedicated clothes and boots for each animal sector?

6) Is there a specific cleaning protocol during sanitary stop?

7) Which type of floor is present in the different productive sectors?

8) Where is the carcasses’ refrigerated room?

Questionnaire S1. Questionnaire administered to swine farmers to collect information about biosecurity and general management of the farm; northern Italy, 2019-2020.
